# Supplementary material for: Myopalladin knockout mice develop cardiac dilation and show a maladaptive response to mechanical pressure overload
Source: eLife. 2021 Sep 24;10:e58313. doi: 10.7554/eLife.58313 (PMC8547954; doi:10.7554/eLife.58313)
Supplement: Figure 3—source data 1. [file elife-58313-fig3-data1.docx]

**Figure 3–source data 1:** Echocardiographic parameters of male WT and MKO male mice at different ages.

|  | **10W** | | **4M** | | **6M** | |
| --- | --- | --- | --- | --- | --- | --- |
|  | **WT (n = 18)** | **MKO (n = 23)** | **WT (n = 43)** | **MKO (n = 27)** | **WT (n = 16)** | **MKO (n = 18)** |
| **Age (weeks)** | 9.7 ± 0.2 | 9.3 ± 0.1 | 17.4 ± 0.1 | 17.6 ± 0.1 | 26.2 ± 0.1 | 26.7 ± 0.2 |
| **BW (g)** | 24.9 ± 0.5 | 23.9 ± 0.3 | 29.4 ± 0.3 | 26.4 ± 0.3^***^ | 30.9 ± 0.7 | 27.9 ± 0.5^***^ |
| **Heart rate (bpm)** | 603 ± 14 | 563 ± 15 | 606 ± 12 | 560 ± 10^*^ | 608 ± 11 | 563 ± 14 |
| **LVIDd (mm)** | 3.40 ± 0.05 | 3.56 ± 0.03^*^ | 3.41 ± 0.03 | 3.87 ± 0.04^***^ | 3.65 ± 0.04 | 3.88 ± 0.05^**^ |
| **LVIDs (mm)** | 2.08 ± 0.04 | 2.26 ± 0.03^**^ | 2.05 ± 0.03 | 2.70 ± 0.04^***^ | 2.29 ± 0.03 | 2.75 ± 0.06^***^ |
| **IVSd (mm)** | 0.78 ± 0.01 | 0.79 ± 0.01 | 0.82 ± 0.01 | 0.83 ± 0.01 | 0.85 ± 0.02 | 0.84 ± 0.02 |
| **IVSs (mm)** | 1.17 ± 0.01 | 1.20 ± 0.02 | 1.25 ± 0.01 | 1.19 ± 0.02^**^ | 1.25 ± 0.01 | 1.21 ± 0.02 |
| **LVPWd (mm)** | 0.77 ± 0.02 | 0.78 ± 0.02 | 0.81 ± 0.01 | 0.83 ± 0.01 | 0.80 ± 0.01 | 0.82 ± 0.02 |
| **LVPWs (mm)** | 1.21 ± 0.01 | 1.18 ± 0.01 | 1.22 ± 0.01 | 1.15 ± 0.02^***^ | 1.23 ± 0.01 | 1.18 ± 0.02^*^ |
| **FS (%)** | 38.9 ± 0.4 | 36.7 ± 0.4^**^ | 40.0 ± 0.4 | 30.2 ± 0.4^***^ | 37.3 ± 0.4 | 29.3 ± 0.6^***^ |
| **EF (%)** | 70.5 ± 0.6 | 67.5 ± 0.6^**^ | 71.7 ± 0.5 | 58.2 ± 0.6^***^ | 68.2 ± 0.6 | 56.8 ± 0.9^***^ |
| **RWT** | 0.456 ± 0.009 | 0.442 ± 0.008 | 0.481 ± 0.007 | 0.430 ± 0.006^***^ | 0.453 ± 0.007 | 0.429 ± 0.011 |
| **CO** | 19.6 ± 0.6 | 20.0 ± 0.5 | 20.5 ± 0.4 | 21.0 ± 0.5 | 23.4 ± 0.7 | 20.9 ± 0.8^*^ |
|  | **WT (n = 12)** | **MKO (n = 24)** | **WT (n = 17)** | **MKO (n = 14)** | **WT (n = 20)** | **MKO (n = 11)** |
| **BW (g)** | 24.7 ± 0.5 | 22.3 ± 0.3^**^ | 29.0 ± 0.5 | 25.6 ± 0.6^***^ | 31.7 ± 0.6 | 26.9 ± 0.7^***^ |
| **HW (mg)** | 125 ± 3 | 116 ± 2 | 136 ± 4 | 133 ± 4 | 154 ± 5 | 140 ± 2^*^ |
| **HW/BW (mg/g)** | 5.05 ± 0.08 | 5.19 ± 0.08 | 4.70 ± 0.10 | 5.20 ± 0.09^**^ | 4.88 ± 0.13 | 5.21 ± 0.10 |

WT, wildtype; MKO, MYPN knockout; W, weeks; M, months; LVID, left ventricular inner diameter; IVS, interventricular septum; LVPW, left ventricular posterior wall thickness; FS, fractional shortening; EF, ejection fraction; RWT, relative wall thickness; CO, cardiac output; BW, body weight; HW, heart weight; bpm, beats per minute; d, diastole; s, systole. Data are presented as mean ± SEM. *p < 0.05, **p < 0.01, ***p < 0.01 *vs*. WT; Two-way ANOVA with Bonferroni’s multiple comparisons test.
