# Supplementary material for: Myopalladin knockout mice develop cardiac dilation and show a maladaptive response to mechanical pressure overload
Source: eLife. 2021 Sep 24;10:e58313. doi: 10.7554/eLife.58313 (PMC8547954; doi:10.7554/eLife.58313)
Supplement: Figure 4—source data 1. [file elife-58313-fig4-data1.docx]

**Figure 4–source data 1:** Echocardiographic parameters of 8-week-old male wild-type (WT) and myopalladin knockout (MKO) male mice before and after transaortic constriction (TAC).

|  | **Before TAC** | | **TAC 1W** | | **TAC 2W** | | **TAC 4W** | |
| --- | --- | --- | --- | --- | --- | --- | --- | --- |
|  | **WT**  **(n = 17)** | **MKO**  **(n = 19)** | **WT**  **(n = 15)** | **MKO**  **(n = 21)** | **WT**  **(n = 8)** | **MKO**  **(n = 9)** | **WT**  **(n = 8)** | **MKO**  **(n = 11)** |
| **Age (weeks)** | 10.1 ± 0.2 | 10.0 ± 0.2 | 11.0 ± 0.3 | 11.4 ± 0.2 | 11.9 ± 0.1 | 11.8± 0.1 | 14.1 ± 0.1 | 14.2 ± 0.2 |
| **Body weight (g)** | 25.4 ± 0.4 | 23.5 ± 0.4^**^ | 25.2 ± 0.5 | 23.9 ± 0.4 | 27.0 ± 0.4 | 24.2 ± 0.6^*^ | 28.5 ± 0.6 | 26.5 ± 0.4 |
| **Heart rate (bpm)** | 627 ± 8 | 588 ± 13 | 594 ± 14 | 558 ± 13 | 584 ± 14 | 581 ± 10 | 574 ± 20 | 563 ± 17 |
| **LVIDd (mm)** | 3.36 ± 0.04 | 3.47 ± 0.03 | 3.37 ± 0.05 | 3.90 ± 0.05^***^ | 3.53 ± 0.08 | 4.16 ± 0.08^***^ | 3.75 ± 0.07 | 4.71 ± 0.11^***^ |
| **LVIDs (mm)** | 2.04 ± 0.04 | 2.13 ± 0.03 | 2.24 ± 0.04 | 2.88 ± 0.07^***^ | 2.35 ± 0.08 | 3.27 ± 0.13^***^ | 2.64 ± 0.07 | 3.82 ± 0.13^***^ |
| **IVSd (mm)** | 0.80 ± 0.01 | 0.78 ± 0.02 | 0.89 ± 0.02 | 0.89 ± 0.02 | 0.86 ± 0.02 | 0.82 ± 0.01 | 0.86 ± 0.02 | 0.82 ± 0.02 |
| **IVSs (mm)** | 1.17 ± 0.02 | 1.18 ± 0.02 | 1.26 ± 0.02 | 1.25 ± 0.02 | 1.25 ± 0.04 | 1.15 ± 0.03 | 1.26 ± 0.02 | 1.14 ± 0.02^**^ |
| **LVPWd (mm)** | 0.77 ± 0.01 | 0.76 ± 0.02 | 0.95 ± 0.02 | 0.96 ± 0.02 | 0.94 ± 0.02 | 0.93 ± 0.02 | 0.95 ± 0.02 | 0.92 ± 0.03 |
| **LVPWs (mm)** | 1.19 ± 0.01 | 1.18 ± 0.01 | 1.36 ± 0.02 | 1.32 ± 0.02 | 1.35 ± 0.02 | 1.22 ± 0.02^***^ | 1.35 ± 0.01 | 1.21 ± 0.04^*^ |
| **FS (%)** | 39.2 ± 0.6 | 38.8 ± 0.5 | 33.5 ± 0.5 | 26.3 ± 0.9^***^ | 33.6 ± 0.9 | 21.7 ± 1.9^***^ | 29.6 ± 0.8 | 19.0 ± 1.4^***^ |
| **EF (%)** | 70.8 ± 0.7 | 70.2 ± 0.6 | 63.4 ± 0.8 | 52.0 ± 1.5^***^ | 63.3 ± 1.3 | 43.9 ± 3.3^***^ | 57.3 ± 1.3 | 38.9 ± 2.5^***^ |
| **RWT** | 0.47 ± 0.01 | 0.44 ± 0.01 | 0.55 ± 0.02 | 0.48 ± 0.01^**^ | 0.51 ± 0.02 | 0.42 ± 0.01^**^ | 0.48± 0.01 | 0.37 ± 0.01^***^ |
| **CO** | 20.6 ± 0.7 | 20.5 ± 0.5 | 17.6 ± 0.7 | 19.1 ± 0.7 | 19.1 ± 1.0 | 19.3 ± 1.1 | 19.9 ± 1.2 | 22.3 ± 1.3 |
|  |  |  |  |  |  |  | **WT**  **(n = 10)** | **MKO**  **(n = 16)** |
| **BW (g)** |  |  |  |  |  |  | 28.3 ± 0.5 | 24.7 ± 0.4^***^ |
| **HW (mg)** |  |  |  |  |  |  | 190 ± 7 | 206 ± 13 |
| **HW/BW (mg/g)** |  |  |  |  |  |  | 6.70 ± 0.18 | 8.30 ± 0.47^**^ |

WT, wildtype; MKO, MYPN knockout; W, weeks; TAC, transverse aortic constriction; LVID, left ventricular inner diameter; IVS, interventricular septum; LVPW, left ventricular posterior wall thickness; FS, fractional shortening; EF, ejection fraction; RWT, relative wall thickness; CO, cardiac output; BW, body weight; HW, heart weight; bpm, beats per minute; d, diastole; s, systole. Data are presented as mean ± SEM. *p < 0.05, **p < 0.01, ***p < 0.01 *vs*. WT; LMM with Bonferroni’s multiple comparisons test.
