# Supplementary material for: Myopalladin knockout mice develop cardiac dilation and show a maladaptive response to mechanical pressure overload
Source: eLife. 2021 Sep 24;10:e58313. doi: 10.7554/eLife.58313 (PMC8547954; doi:10.7554/eLife.58313)
Supplement: Supplementary file 1. [file elife-58313-supp1.docx]

**Supplementary file 1.** Oligos used for qRT-PCR and clonings

| **Primers** | **Sense** | **Reverse** |
| --- | --- | --- |
| **qRT-PCR (mouse)** |  |  |
| *Mypn* | CATGCTTTGCTTCCAACATT | GGCTTCTGGATTCGATTCAT |
| *Palld 200 kDa* | CATCCAGAAACTGAGGAGCC | AGCTTTCGCTGTCAGAGTCC |
| *Palld 140 kDa* | TGCTGCCTGTGCATTTTCCC | AGCTTTCGCTGTCAGAGTCC |
| *Palld 90 kDa* | AGGAGCCCTCGACACCCA | TCCTGTTCCAGGCGCACTTGG |
| *Palld all isoforms* | GTATTGCCACCAACAGAG | CCTTGTGTGCTTCCTTAG |
| *Ankrd1* | GCTGGAGCCCAGATTGAA | CTCCACGACATGCCCAGT |
| *Nppa* | ATCTGCCCTCTTGAAAAGCA | ACACACCACAAGGGCTTAGG |
| *Nppb* | GGACCAAGGCCTCACAAAAG | AAAGAGACCCAGGCAGAGTC |
| *Acta1* | AATGAGCGTTTCCGTTGC | ATCCCCGCAGACTCCATAC |
| *Actc1* | TGCCGATCGTATGCAAAAGG | GGCCTGCCTCATCATACTCT |
| *Myh6* | CGCATCAAGGAGCTCACC | CCTGCAGCCGCATTAAGT |
| *Myh7* | GCATCAAGGAGCTCACC | CTGCAGCCGCAGTAGGTT |
| *Casq2* | CCGCACGATTGAGTTTGAC | CACGATCTCCACTGGGTCTT |
| *B2m* | CCGTCTACTGGGATCGAGAC | GCTATTTCTTTCTGCGTGCAT |
| **Clonings** | | |
| pET-3d-6xHis human MYPN C-term. (Res. 813-1320; NM_032578.3) | ggctcgagt/TCCCCAATTCCTGTCTCTC | ggggaattc/TTAAAGTTCATCACTCT |
| pETM-14 human MYPN C-term.  (Res. 945-1320; NM_032578.3) | ttccaggggcccatg/CCCATCTTTGACAAGAGACTCA | ggtggtggtgctcga/TTAAAGTTCATCACTCTCCACTAC |
| pET-3d-6xHis human Titin Z4-Z5  (Res. 942-1173; NM_001256850.1) | gggggctcgagatg/CCACCAACTTTGGTCTCGGGC | gggggaattctca/TTCAAGCAAGGAAGCAGATGCAGA |
| pETM-14 human PALLD C-term.  (Res. 794-1123; NM_001166108.1) | ttccaggggcccatg/CCATTCTTTGAGATGAAGCTGAAAC | ggtggtggtgctcga/TTAATGTCGAGAAATGTAAACGTCCAG |
| pGBKT7 human Titin IgZ4-Z5  (Res. 942-1173; NM_001256850.1) | catggaggccgaattc/CCACCAACTTTGGTCTCGGGC | gcaggtcgacggatcctca/TTCAAGCAAGGAAGCAGATGCAGA |
| pGADT7 human MYPN full-length (Res. 1-1320; NM_032578.3) | ggaggccagtgaattc/ATGCAAGACGACAGCATAGAAGCTTCTACT | cgagctcgatggatcc/TTAAAGTTCATCACTCTCCACTACACTCCG |
| pGADT7 human MYPN Ig3-end  (Res. 945-1320; NM_032578.3) | aactcgag/AGCCCATCTTTGACAAGAGACTC | ggggaattc/TTAAAGTTCATCACTCTCCACTAC |
| pGADT7 human MYPN Ig5-end  (Res. 1172-1320; NM_032578.3) | ctcgag/AGCCTGTGATCCTGGAGAAAC | ggggaattc/TTAAAGTTCATCACTCTCCACTAC |
| pGADT7 human MYPN Ig3-4  (Res. 945-1171; NM_032578.3) | aactcgag/AGCCCATCTTTGACAAGAGACTC | ggggaattc/TTATGCTTTCTTCACCTCTTTGGC |
| pGADT7 human MYPN Ig4-end  (Res. 1073-1320; NM_032578.3) | aactcgag/AGCCACATTTCCTGCAGGCTC | ggggaattc/TTAAAGTTCATCACTCTCCACTAC |
| pGADT7 human PALLD C-term.  (Res. 794-1123; NM_001166108.1) | ggaggccagtgaattc/CCATTCTTTGAGATGAAGCTGAAAC | cgagctcgatggatcc/TTAATGTCGAGAAATGTAAACGTCCAG |
| pLexA titin Z3-Z5 (Res. 3052-4371; NM_001256850.1) | tttctcgagc/CCAACTTTGGTCTCGGGCTTA | tttggatcccta/CTCCACATACAATTTCCCTGA |
| pLexA titin Z4-Z5 (Res. 3452-4371; NM_001256850.1) | tttctcgagc/CCTGGAGAACCTGCCGCGCC | tttggatcccta/CTCCACATACAATTTCCCTGA |

Vector specific sequence is written in lowercase letters. FL, full-length
